# Cell Groups Reveal Structure of Stimulus Space

**DOI:** 10.1371/journal.pcbi.1000205

**Published:** 2008-10-31

**Authors:** Carina Curto, Vladimir Itskov

**Affiliations:** 1 Center for Molecular and Behavioral Neuroscience, Rutgers, The State University of New Jersey, Newark, New Jersey, United States of America; 2 Center for Theoretical Neuroscience, Columbia University, New York, New York, United States of America; University College London, United Kingdom

## Abstract

An important task of the brain is to represent the outside world. It is unclear how the brain may do this, however, as it can only rely on neural responses and has no independent access to external stimuli in order to “decode” what those responses mean. We investigate what can be learned about a space of stimuli using only the action potentials (spikes) of cells with stereotyped—but unknown—receptive fields. Using hippocampal place cells as a model system, we show that one can (1) extract global features of the environment and (2) construct an accurate representation of space, up to an overall scale factor, that can be used to track the animal's position. Unlike previous approaches to reconstructing position from place cell activity, this information is derived without knowing place fields or any other functions relating neural responses to position. We find that simply knowing which groups of cells fire together reveals a surprising amount of structure in the underlying stimulus space; this may enable the brain to construct its own internal representations.

## Author Summary

We construct our understanding of the world solely from neuronal activity generated in our brains. How do we do this? Many studies have investigated how neural activity is related to outside stimuli, and maps of these relationships (often called receptive fields) are routinely computed from data collected in neuroscience experiments. Yet how the brain can understand the meaning of this activity, without the dictionary provided by these maps, remains a mystery. We tackle this fundamental question in the context of hippocampal place cells—i.e., neurons in rodent hippocampus whose activity is strongly correlated to the animal's position in space. We find that the structure of stimulus space can be revealed by exploiting relationships between groups of cofiring neurons in response to different stimuli. We provide a ‘proof of principle’ by demonstrating constructively how the topology of space and the animal's position in an environment can be derived purely from the action potentials fired by hippocampal place cells. In this way, the brain may be able to build up structured representations of stimulus spaces that are then used to represent external stimuli.

## Introduction

Stimulus reconstruction, as implemented by the scientist, typically involves three steps: (i) characterizing the space of relevant stimuli; (ii) constructing functions relating stimuli to neuronal responses; and (iii) using these functions, together with new neuronal activity, in order to “decode” new stimuli [1]–[11]. For example, in the case of hippocampal place cells, the ‘space of stimuli’ may be the animal's current spatial environment; for every place cell one computes a *place field*, i.e., a function that assigns a firing rate to each position in space. Place fields, together with place cell activity, can then be used to infer the animal's position [2],[4],[7]. Notably, the scientist relies on a priori assumptions about the nature of the relevant stimulus space in (i), and uses *independent measurements* of previously observed stimuli in order to construct the functions in (ii). While these functions (or “neural codes”) come in a variety of forms, such as receptive fields, tuning curves, spike-triggered averages, adaptive filters and conditional probability distributions [1]–[11], they all require using independent observations of prior stimuli for their construction.

Presumably, the brain also uses neuronal spiking activity to reconstruct the stimulus. The brain, however, does not have access to independent stimulus measurements; neuronal activity alone must represent the external world. How does the brain do it? While much effort has been devoted to developing biologically plausible methods to implement the “decoding” of step (iii) [1],[3],[6],[10],[11], it is generally assumed that the structure of stimulus space (step (i)) and the functions (such as receptive fields or tuning curves) of step (ii) are both present and easily available to downstream structures in the brain. Although it is possible that receptive fields are imprinted in synaptic weights, tuned throughout development and learning, this story is complicated by the observation that receptive fields in some brain areas—particularly in hippocampus—undergo rapid context-dependent remapping [2], [12]–[19]. This leads naturally to the question: can anything be inferred about a stimulus from spikes alone?

We address this question in the context of hippocampal place cells. In rodents, spatial information is reflected in the activity of place cells, i.e., pyramidal cells in areas CA1 and CA3 of dorsal hippocampus that fire in a restricted area of the spatial environment—the place field—and are mostly silent outside [20],[21]. We will use the term *place field* to refer both to the function and to the region in space where the firing rates are significantly above baseline. Place fields remap, and a place cell can alternate between multiple stable place fields as an animal is switched from one familiar environment to another [14],[15]. Although much work has gone into trying to understand how place fields are formed [13], [22]–[25], a different and rather unexplored question is how the output of hippocampal place cells (*without* access to corresponding place fields) might be used by downstream structures in order to reconstruct position and the underlying space.

At first glance, it is not obvious that anything at all may be learned about a particular environment—or the animal's position within it—using the spiking activity of place cells alone. Indeed, previous approaches to reconstructing position from place cell activity have all required knowing the corresponding place fields [2],[4],[7]. Furthermore, the values of instantaneous firing rates [2],[4] and the precise timing of spikes with respect to the theta rhythm [7] have been used, together with place fields, in order to improve position-estimation precision beyond the place field diameter. It has also been suggested that other cell types, such as head direction cells, play a vital role in deciphering position information [22]. Because place fields exhibit complex dynamics and place cells do more than just coding for place [26]–[30], it is important to identify—at least in theory—minimal aspects of neural activity that yield sufficient information for construction of an accurate representation of space.

In this work we show that a great deal of information about a physical environment can be obtained using only very coarse features of population spiking activity. We define a ‘cell group’ as a collection of cells that collectively fire significantly above baseline within a broad (∼250 ms) temporal window; we do not call them ‘cell assemblies’ to avoid confusion with different timescales and degrees of sensory control implied by this term [31]–[34]. We find that the simple knowledge of which groups of hippocampal place cells fire together is enough to (1) extract global topological features of the environment, and (2) reconstruct an accurate geometric representation of physical space within which the animal's position can be faithfully tracked. This is made possible by using standard tools from algebraic topology and graph theory; neither place fields, nor precise spike timing, nor any prior independent measurements of position are needed.

## Results

### Cell Groups Reveal Place Field Intersection Information

Although the brain may be unable to establish direct relationships (such as place fields) between neural responses and external stimuli, it *can* in principle compare neural responses to each other. Moreover, relationships between neural responses reflect relationships between stimuli, and hence reveal structure of the outside world.

**Figure 1:**
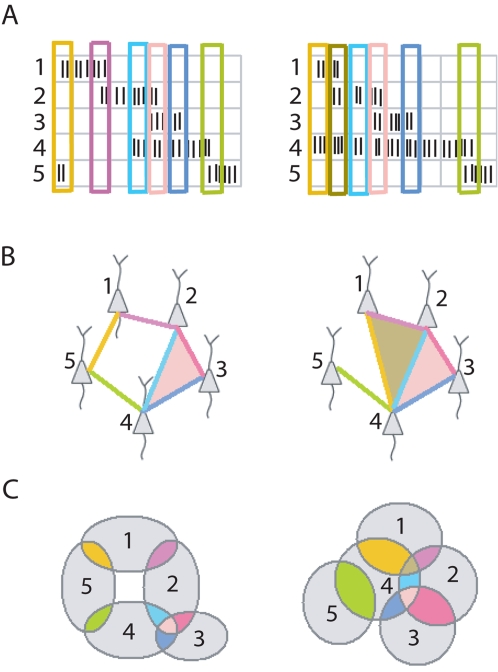
Collection of cell groups uniquely determines the topology of the environment. (A) Sample rasters for the population activity of five place cells in two different environments. Cell groups are obtained by identifying subsets of cells that co-fire within a coarse time window (colored rectangles). (B) Two examples of five-cell configurations (simplicial complexes) depicting collections of cell groups obtained from the sample rasters in (A). An edge represents a cell group with two cells and a shaded triangle indicates a cell group with three cells; colors correspond to cell groups in (A). (C) Cells that co-fire have overlapping place fields. Each cell group in (A), (B) corresponds to a particular intersection of place fields, denoted with matching color. The place field intersection pattern fully determines the topology of a space covered by convex place fields. The first configuration in (B) forces an arrangement of place fields with a hole in the middle (left); the second forces a space with no holes (right).

In rat hippocampus, the theta-oscillation (6–10 Hz) provides a natural timescale for organizing population activity. Cells that fire within a few theta-cycles of each other are very likely to have overlapping place fields. We define a *cell group* as a group of place cells that collectively fire within a two theta-cycle (250 ms) time window (Figure 1A). Note that this enables us to ignore finer spike timing effects modulated by the phase of the theta oscillation, such as phase precession [7], [31], [35]–[37]. Each place cell typically belongs to multiple cell groups (Figure 1B), and the activation of a given cell group is induced by the animal passing through the intersection of corresponding place fields. Cell groups thus yield place field *intersection information* (i.e., they reveal which subsets of place fields overlap), even when the place fields themselves are unknown (Figure 1C).

We first show that this intersection information can be patched together to reveal global topological features of the environment. The method for extracting global topological features does *not* require a metric. On the other hand, by thinking of each cell group as defining a specific location in space, we can use intersection information to construct a metric that provides relative distances between cell groups. This yields a geometric representation of the external physical space, obtained without knowing place fields. We find that this internal representation is quite faithful to the geometry of the environment. In either case, we need only make some basic assumptions about place fields. We assume that place fields exist and are stable, have similar sizes, are omni-directional, and have firing fields that are convex. These assumptions are generally satisfied for place fields of dorsal hippocampal place cells recorded from a freely foraging rat in a familiar open field environment (see Methods). We also explicitly test the importance of the assumption that place fields have similar sizes, and find that our results are in fact fairly robust to substantial variability in place-field sizes. Finally, we test our methods with multipeaked place fields, and find that our algorithms can tolerate a realistic percentage of cells having multiple firing fields, so long as the component fields are sufficiently separated and convex.

### Global Topological Features

What may be thought of as a ‘space of stimuli’ at one level of processing may constitute an individual stimulus at another: global features of the ‘space of positions’ become properties of individual environments that can be used to distinguish between them. Often times an animal's physical space has “holes”—i.e., regions in the interior of the environment where the animal is unable to go. For example, a rat may be confined to a platform with one or more holes in the middle; similarly, there may be large objects inside the environment (such as trees) providing obstructions to the animal's path. In either case, we call the region inaccessible to the animal a *hole*.

Holes are examples of (non-metric) topological features, because they are preserved under continuous deformations of the space. Two environments are said to be *topologically equivalent* (homeomorphic) if one can be continuously deformed into the other, and vice versa. *Homology groups* [38] (see Text S1) are topological invariants that can be used to distinguish topologically inequivalent spaces. In particular, the dimension of the first homology group H_1_ counts the number of holes. Higher order homology groups (H_2_, H_3_, …) count higher-dimensional “holes,” and thus place constraints on the minimum dimensionality of the space; they are all expected to vanish for flat, two-dimensional environments.

### Topological Features Can Be Extracted from Cell Groups

From spike trains for a population of place cells, we obtain a collection of cell groups (Figure 1A; see also Methods). The corresponding intersection information can be used to compute homology groups of the underlying environment—even though the place fields themselves are unknown. Intuitively, this works because there is a unique configuration of place fields (up to continuous deformation) consistent with a given pattern of intersections (Figure 1B and 1C). Inspired by a deep theorem in algebraic topology [38] (see Text S1), we have devised a procedure to compute homology groups from the collection of cell groups active in a given environment. This theorem has also been used in the context of sensor networks [39], and the potential utility of similar methods in the case of hippocampal place cells was independently observed in [40]. Our algorithm, described in detail in the Methods, involves constructing a simplicial complex (Figure 1B) from place field intersection information, and computing its homology groups. If the cell groups obtained from spike train data exactly reflect the correct place field intersection information, the theorem guarantees that the homology of the simplicial complex is equal to the homology of the underlying space. However, given the stochastic nature of place cell firing, there is always the possibility that we may miss cell groups corresponding to real intersections, and mistakenly detect cell groups corresponding to non-existent place field intersections.

**Figure 2:**
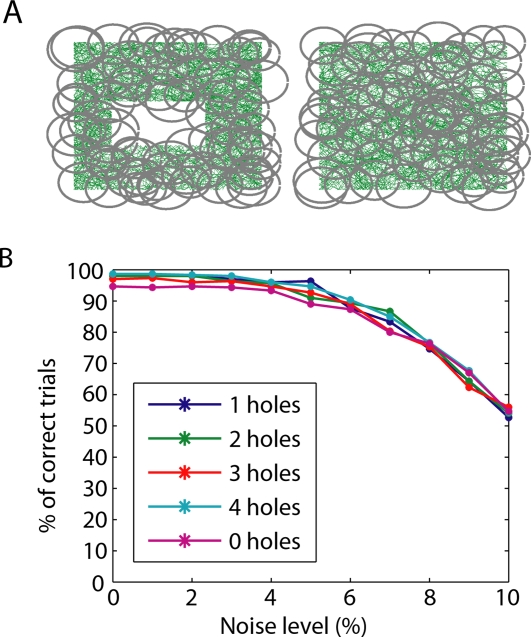
Accuracy of extracted topological features. (A) Sample trajectories (green) in environments with one and zero holes. Gray circles depict place fields used to simulate data for one trial. (B) For each environment, and for each level of added noise, the percentage of correct trials was computed from 300 trials (each having a different set of randomly-generated place fields). A trial was considered ‘correct’ if all five computed homology groups matched the topology of the environment, and ‘incorrect’ if at least one homology group did not match.

In order to verify that this procedure yields accurate results within physiologically realistic parameters, we tested it using simulated data with varying degrees of noise. Random-walk trajectories were generated in five different flat, two-dimensional environments, each of side length *L* (typically *L*∼1 m), with *N* = 0,1,…,4 holes (Figure S1). In each of 300 trials, each of the five environments was covered by 70 single-peaked place fields with varying radii (0.1–0.15 *L*) and randomly-chosen centers (Figure 2A). Place cell firing was generated according to a simple model (see Methods). Differing levels of noise were introduced by removing a certain percentage of randomly-selected spikes from each spike train (for every cell) and reassigning them to occur at random times, so that they fall outside the place field (see Methods).

For each trial, the first five homology groups (H_0_,…,H_4_) were computed. A trial was deemed to be ‘correct’ if and only if all homology groups matched the topology of the underlying space, and ‘incorrect’ if at least one homology group did not match. Although the correct environment could be identified using only the first homology group H_1_, we required the other homology groups to also match in order to ensure consistency of the overall topology (i.e., this was not a multiple-choice framework where each trial was assigned the ‘most likely’ of the five environments; note that ‘chance level’ here is close to 0%). For low levels of noise, we found near 100% accuracy in all environments (Figure 2B).

One might worry that if a cell ever spikes outside of its place field, it will activate a cell group that does not correspond to a true intersection of place fields, rendering the topology computations completely inaccurate. Remarkably, the percentage of correct trials remained very high for noise levels up to 5%; even with 10% of each cell's spikes occurring outside the corresponding place field, more than half of all trials continued to be correct (Figure 2B). This is because the thresholding of firing rates in order to obtain cell groups (see Methods) renders the procedure quite robust to noise in the spike trains. Note that although the trajectory of the rat is itself littered with small holes (Figure 2A), the procedure only detects actual (relatively large) holes in the environment and is quite insensitive to holes that are very small compared to the sizes of place fields.

As a further test, we constructed additional ‘shuffled’ data sets by pooling together spiking activity from cells in different environments. We found that each and every ‘shuffled’ trial had nonzero higher homology groups, suggesting higher-dimensional spaces. This indicates that population activity in the shuffled data sets was not generated from realistic, two-dimensional environments, and suggests that a downstream structure receiving hippocampal output could detect patterns of activity that are inconsistent with a spatial interpretation.

### An Internal Representation of Space Can Be Built from Cell Groups

**Figure 3:**
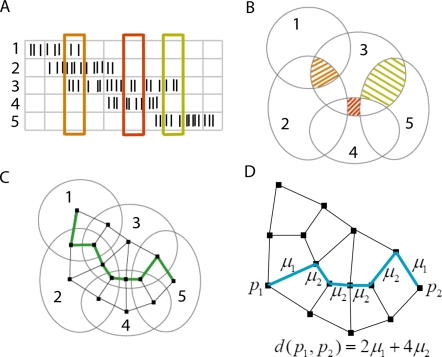
Construction of a metric on cell groups. (A) Example spike trains from five place cells. Each time bin (columns) represents two theta cycles. (B) Place field intersection pattern derived from cell groups in (A). Shaded regions correspond to cell groups inside rectangles of the same color in (A). (C) The pattern of intersections can be represented by a graph, with vertices (black squares) for each cell group, and edges connecting neighbors (cell groups that differ by one cell only). A trajectory (green) is inferred from the example data, by “connecting the dots” to match the sequence of cell groups in (A). (D) Weights are assigned to edges of the graph using the dissimilarity index *μ_k_*, where *k* is the number of common cells between neighbors. The distance between any two vertices in the graph is obtained by summing the weights along a shortest path (blue).

A given cell group becomes active when the animal crosses a specific location in space, given by the intersection of the corresponding place fields. It is thus natural that, from the brain's point of view, a location in space is itself *defined* by a cell group (Figure 3A and 3B). The collection of all activated cell groups thus yields a collection of points, which can be thought of as “building blocks” for an internal, discretized representation of space. A set of unrelated points, however, does not constitute a space, one must know the relationships between points (which pairs are close, and which are far away). Fortunately, there is a natural way to determine when two cell groups are “close” to each other, based on the number of place cells they have in common.

We say that two cell groups are *neighbors* if they differ by just one place cell. By joining neighboring points with edges, one obtains a graph (Figure 3C) that is constructed purely from cell groups, without any explicit knowledge of place fields. In general, neighboring cell groups with a very high percentage of overlapping cells will represent points that are closer in space than neighbors with small overlap. We define a *dissimilarity index μ_k_* on neighboring cell groups as the average relative distance between the centers of adjacent regions with overlap degree *k*, assuming place fields of equal radius (see Methods). In principle, *μ_k_* should be derivable from basic geometry, as it depends only on general and unchanging properties of physical space. We estimated *μ_k_* empirically by computing the average distances between the centers of adjacent intersection regions for 30 randomly-generated sets of place fields covering the environment, and normalized the index by fixing the largest value *μ*_1_ = 1 (see Methods). We found that for 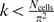, the index is well approximated by the formula 
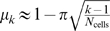
, where *N*_cells_ is the number of place cells active in the environment (see Figure S2). We assume such an index can be “hard-wired” in the brain, as it has no information about any particular arrangement of place fields or any particular environment. Although we estimated *μ_k_* assuming all place fields have identical size, we use exactly the same formula for *μ_k_* in every reconstruction, regardless of the distribution of place field sizes we consider.

The dissimilarity index can be used to assign weights to each edge in the graph. A *path* is a sequence of edges connecting two vertices (cell groups) in the graph; the length of a particular path is given by summing the weights along its edges. The distance between any two cell groups in the graph can then be defined as the length of a shortest path between those points (Figure 3D; see also Methods). In this manner one obtains a natural *metric* on cell groups. We call this graph, with cell groups as its vertices and edges between neighbors, together with the metric, the *internal representation* of the external space.

### Internal Representation Accurately Reflects External Geometry

In order to test how well the internal representation conforms to the geometry of the external space, we used simulated population spiking activity from a two-dimensional square box environment (see Methods) with differing numbers of place cells. For each number of place cells covering the environment, we randomly generated data sets for 60 trials, each trial having different place fields of radii chosen uniformly at random from the interval [0.1 *L*,0.125 *L*], with randomly-chosen centers. The place field sizes were chosen to conform to the 20–25 cm range of average diameters typically observed for place cells in dorsal hippocampus for a rat exploring an open field environment of scale *L*∼1 m [41],[42]. For each simulated data set, we constructed an internal representation as outlined above.

**Figure 4:**
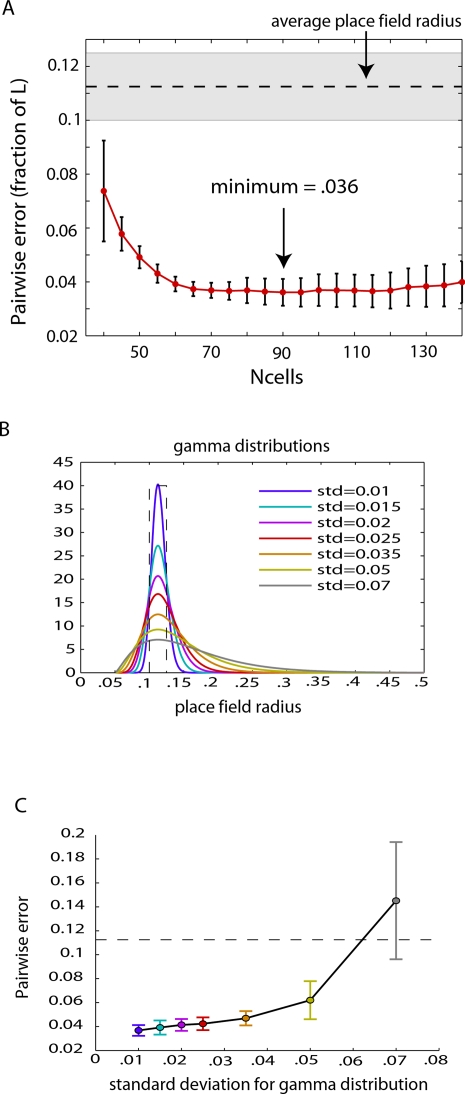
Error in pairwise distances computed from internal geometric representation of space. (A) For a fixed number of place cells, the pairwise error was computed and averaged over 60 trials. Each data set was generated from a different set of place fields, with randomly selected centers and radii chosen uniformly at random from the interval [0.1,0.125] (shaded gray region; this corresponds to place field diameters ranging from 20–25 cm in a 1 m×1 m environment). The dashed horizontal line corresponds to the average radius of place fields. The average pairwise error achieved a minimum of 0.036 (as a fraction of box side length *L*) for 90 cells, and then leveled off. This indicates that relative distances between pairs of points in the internal representation are accurate to within an error that is less than 1/3 the average radius of place fields. (B) Various gamma-function distributions for place field sizes, with fixed mode = 0.1125 *L* and varying standard deviations. Radii greater than 0.5 *L* are not considered, as these correspond to place field diameters that exceed the side length of the box. Dashed line indicates the uniform distribution used in (A). (C) Mean pairwise error averaged over 60 trials for *N*_cells_ = 90 and for each of the distributions of place field radius displayed in (B). Dashed line denotes the place field radius corresponding to the peak of each distribution. Error bars in both (A) and (C) represent standard deviations across trials.

To assess the accuracy of the internal representations, we first computed pairwise distances between points on a fine grid spanning the *L*×*L* environment and compared them with the corresponding pairwise distances of their images in the internal representation (Figure S3). We defined the *pairwise error* for an individual trial (having a fixed number of place cells) as the mean error in pairwise distances when computed using the internal representation (see Methods). We found that the average pairwise error across trials had a minimum value of 0.036 *L* for 90 cells (Figure 4A), or less than 1/3 the average place field radius; this indicates that relative distances between points in the internal representation are accurate to within a ball of approximately 1/9 the median place field area. To check robustness of this procedure in the case of greater place field variability, we repeated this analysis for a series of gamma-function distributions of place field radii (Figure 4B). We found that performance decreased slowly for distributions with increasing standard deviations up to ∼0.035, and rapidly deteriorated for distributions with standard deviations greater than 0.05 (Figure 4C).

As a further test that the full geometry—and not just pairwise distances—is accurately reflected in the internal representation, we used multi-dimensional scaling (MDS) [43] to embed each graph into a two-dimensional Euclidean space, in a way that best preserves the relative distances between pairs of points (i.e., to best preserve the metric on cell groups). Next, we “aligned” the coordinates of the embedded internal space properly so as to best match the particular coordinates used to represent the external space (Figure S4; see also Methods). Points in the external space could then be mapped into the embedded internal space by identifying corresponding cell groups (see Methods).

**Figure 5:**
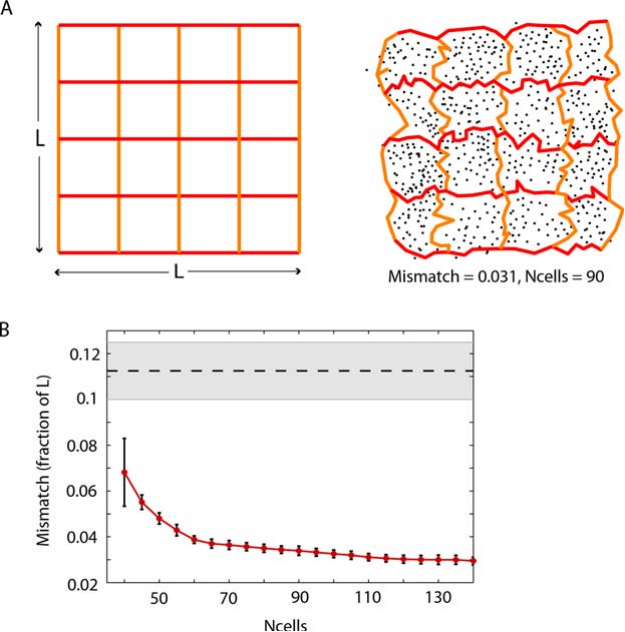
Accuracy of full geometry for internal representation of space. (A) The original space (left) and a reconstruction from simulated place cell activity (right). Black dots correspond to cell groups. A coarse grid (red and orange lines) in the original space is mapped into the reconstructed space, to allow for visual comparison of the geometry. (B) The accuracy of a reconstructed space may be quantified by computing the ‘mismatch’ between points in the original space and their images in the reconstructed space, as a fraction of the box side length *L*. The mismatch decreases with increasing number of cells. Error bars correspond to standard deviations for average mismatch across 60 trials. The dashed horizontal line corresponds to the average radius of place fields, while the shaded gray area corresponds to the range of place field radii.

Visually, the quality of an internal representation can be judged by mapping a coarse grid of vertical and horizontal lines from the external space into the embedded internal space, and seeing how faithfully the geometric structure is preserved. We found that the full metric geometry (including angles and relative distances) of the internal representation closely mirrored that of the external space (Figure 5A). In particular, the square shape of the box and the rectilinear structure of the grid were faithfully reproduced. We quantified the accuracy of a given internal representation by computing the *mismatch* between the two spaces; this measure computes the average error, as a fraction of box side length *L*, obtained by mapping a fine grid of points from the original space into the aligned embedded internal space (see Methods). Quite similarly to the pairwise error, we found that the average mismatch decreased with increasing numbers of cells, getting as low as 3% for 120–140 cells (Figure 5B).

### Multipeaked Place Fields

Until now we have assumed that place fields are convex; while this is usually the case, multipeaked place fields are often observed. In open field environments of size *L*∼1 m, a small percentage (5–10%) of place cells have two disconnected firing fields, each of which looks like a convex, single-peaked place field [21],[44]. We will refer to these neurons as “multipeaked place cells,” and to the connected components of the place fields simply as “fields.” At first glance, the presence of multipeaked place cells poses a potential limitation to our study. The algebraic topology theorem no longer holds, suggesting that the algorithm we have thus far used for extracting topological features is likely to fail. In the case of the geometric reconstruction, on the other hand, we do not necessarily expect multipeaked place fields to pose a problem, so long as the component fields are individually convex. In general, the danger with multipeaked place fields is that distant regions of space may be identified as being the same. In an “across-cell” coding scheme where each neuron represents a distinct location in space, this ambiguity indeed poses serious problems [21]. When locations are represented by cell groups, however, this difficulty is easily overcome. Although the same cell may fire in two locations that are far from each other, cell groups corresponding to these distant regions will generally be very different, as other cells serve to disambiguate position. Because two cell groups are considered neighbors only in the case that they share a majority of cells in common, pairs of cell groups with only one or a few common place cells are guaranteed to represent distant positions in the internal representation.

**Figure 6:**
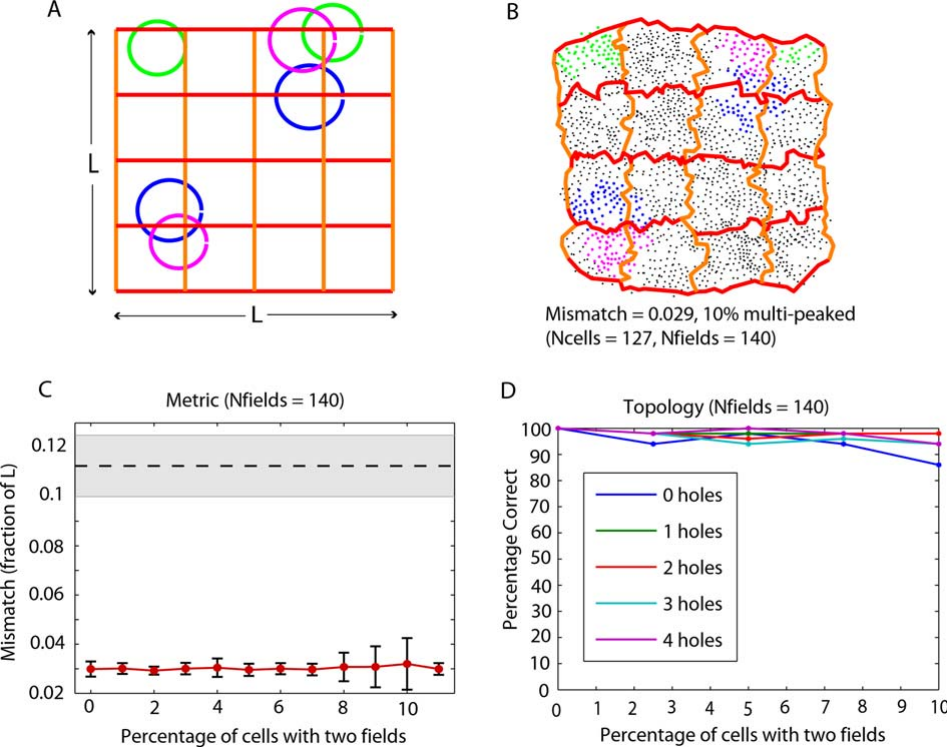
Accuracy of metric reconstructions and topology computations with multipeaked place cells. (A) The original space, together with double-peaked place fields for three example simulated place cells (blue, green and magenta). (B) A reconstructed space obtained from a data set where 10% of the place cells have double-peaked place fields. Black dots correspond to cell groups, as in Figure 5A. Cell groups containing each of the three place cells with multipeaked fields displayed in (A) are shown with corresponding color. Cell groups containing each of the 13 multipeaked place cells in this data set are shown in Figure S5. (C) For a fixed total number of 140 fields covering the environment, the mismatch remains nearly constant for increasing percentages of multipeaked place cells. Error bars correspond to standard deviations for average mismatch across 60 trials. (D) The extraction of topological features also performs well on simulated data including up to 10% multipeaked place cells. The percentage of correct trials was computed across 50 trials with 0%, 2.5%, 5%, 7.5%, and 10% of cells having double-peaked place fields. As in Figure 2B, a trial was considered ‘correct’ if and only if all five computed homology groups matched the topology of the environment.

In order to test the performance of the geometric reconstruction in the case of multipeaked place cells, we simulated data as before but included small percentages (up to 11%) of multipeaked place cells while keeping the total number of firing fields covering the environment constant. In these simulations, we also required that the centers of multiple fields corresponding to the same cell be sufficiently distant; this was in order to enable disambiguation by other cells (see Methods). For 140 fields, we found the performance to be very good (Figure 6). An example reconstructed space from data containing 10% multipeaked place cells demonstrates that the algorithm naturally separates the double fields (Figure 6A and 6B; see also Figure S5). In particular, both the mismatch (Figure 6C) and pairwise error (Figure S6C) remained approximately constant ranging from 0% to 11% multipeaked place cells.

For data generated from only 90 fields, however, as in Figure 5A, performance steadily decreased with increasing numbers of multipeaked place cells, as measured by both pairwise error and mismatch (Figure S6A and S6B). This is because there were not enough place fields to double-cover the environment, leading some regions within double fields to fail to be disambiguated by the presence of other place cells, and thus causing large distortions in the reconstructed space. In fact, this is precisely the problem that may cause the topology algorithm to fail. When the environment is double-covered by place fields, however, place cells with multiple fields can be detected purely from the graph of neighborhood relationships on cell groups (constructed as in Figure 3C). For each place cell, there is an induced subgraph whose vertices are the cell groups containing that cell. Each connected component of this subgraph corresponds to a distinct field (Figure S7). Having detected additional fields for multipeaked place cells, we can then assign auxiliary “place cells” to substitute the original cell labels such that each connected component corresponds to a distinct cell. We then build the simplicial complex and compute homology groups as before. Regarding the (unknown) place fields as providing an open cover of the underlying space, the added step of the topology algorithm can be thought of as making the minimal possible *refinement* [45] of the open cover such that the theorem again holds (see Methods). We found that with this modification, the topology algorithm maintains very good performance for the experimentally observed range of 5–10% multipeaked place cells (Figure 6D).

## Discussion

We have shown that, in the case of hippocampal place cell activity, global topological features of a two-dimensional environment as well as an accurate geometric reconstruction of physical space—including the animal's position within it—can be inferred from spikes alone. In either case, one need only assume that place fields *exist* and have a stereotypical form; knowledge of actual place fields or any other prior independent measurements of position is not needed. This provides a general framework for building up stimulus spaces (or ‘cognitive maps’ [46]) using only neural activity from a relatively homogeneous population of neurons, such as dorsal hippocampal place cells.

Even after obtaining a geometric representation of space, global topological features (if needed) must still be computed. Although we may be able to “see” topological features of the stimulus space by looking at a two-dimensional embedding of the internal representation, this does not mean no further computation is necessary; it merely reflects the fact that our visual system is able to do the computation. Moreover, global features of a ‘space of stimuli’ at one level of processing may become properties of an individual (composite) stimulus at another. Interestingly, although the computation of topological features also has cell groups as its starting point, it does not require constructing a geometric representation of space, and hence bypasses the need for a metric.

At first glance, our internal representation is perhaps reminiscent of the ‘cognitive graph’ in [47],[48], as it is also a graph constructed to represent a physical environment. The ‘cognitive graph,’ however, was envisioned as an actual neural (sub-) network realized in the hippocampus, with an individual place cell for every vertex and a synapse for every edge. The metric was encoded in synaptic weights between place cells, and determined via an LTP learning rule. This implies that geometric distortion would result from a biased sampling of the environment by the animal's trajectory. Although each vertex in the ‘cognitive graph’ was intended to represent the center of the corresponding place field, it is always the case that many place cells are simultaneously active at any given location in space, suggesting that this graph is not suitable to represent specific positions given population place cell activity. Moreover, the existence of multipeaked place cells presents a seemingly insurmountable challenge in this and any paradigm where place cell firing is presumed to signal proximity to a single place [21].

In contrast, our internal representation graph has a vertex for every group of reliably co-firing neurons, and is closer in spirit to Hebb's cell assemblies [34] than to a literal neural network representation of space. Since distances between cell groups result from the combinatorics of their overlaps, the fact that multiple cells co-fire in response to a given stimulus is not a nuisance but a necessary condition for inferring the structure of the underlying space. An essential feature is that, unlike in the case of Kohonen maps [49], geometric relationships between external stimuli are revealed even when neurons in the network have no a priori topographic structure (as in hippocampus). Moreover, the resulting metric on cell groups is insensitive to a biased sampling of the environment or to the particular values of synaptic weights within the hippocampus, and does not depend on nearby stimuli occurring in temporal proximity to each other, as would be required to infer stimulus space structure using spike train metrics [50]. Instead, we have used only a very coarse aspect of population spiking activity—the set of all cell groups—while fully exploiting the fact that co-firing cells have overlapping receptive fields.

These results suggest that it may be possible for maps of the environment to be constructed in downstream brain areas purely from cell groups. If this is the case, we would expect that geometric distortions in the animal's spatial perception would arise as a consequence of uneven place field coverage of an environment: the animal should overestimate distances in a region of higher place field density, and underestimate distances in regions with significantly lower place field density. This prediction, if confirmed by experiment, would provide evidence that only cell groups are used in constructing internal representations of space. If, on the other hand, such perceptual distortions are not observed, we can be almost certain that some other aspect of neural spiking activity contributes. Interestingly, because in our simulations we chose place field *centers* uniformly at random from within the environment, we consistently had a lower density of place field coverage near the boundaries. This, in fact, led to greater geometric distortions near the boundaries of our spatial reconstructions than in the interior. To compensate for this, one might expect there to be a greater number of place fields near the boundary of an environment. Such an effect has, in fact, been reported experimentally [51],[52], and may be regarded as a postdiction.

We have considered environments that are flat and two-dimensional; however, it is easy to generalize our procedures to stimulus spaces that are higher-dimensional and/or curved. Recent experiments suggest that three-dimensional hippocampal place fields may be observable in flying bats [53]. The topology algorithm can be used in exactly the same way to detect holes or obstacles in three-dimensional environments from cell groups. The geometric reconstruction algorithm could also be used in exactly the same manner, the only difference being that a different dissimilarity index *μ_k_*, computed for three-dimensional space, would need to be used. Furthermore, we believe our approach could be generalized to stimulus spaces reflected in other brain areas. The geometric methods could prove useful in discovering new structure in stimulus spaces reflected in neocortical areas, such as primary and higher order sensory cortices. Moreover, the brain appears to be particularly adept at identifying topological properties of complex objects. For example, connected components and holes in a visual object or scene are often among the most salient features. A topological approach, such as the one we have used here, could yield insight into understanding how global features of a visual object are extracted from the activity of cells with spatially localized receptive fields.

Our notion of stimulus reconstruction is a significant departure from traditional “decoding” paradigms, as it does not require directly relating neuronal activity to external stimuli (as in the computation of receptive fields), or to activity in any other area of the nervous system. Moreover, while the computation of receptive fields begins with *a priori* assumptions about the nature of the stimulus space being represented, we recover the structure of the stimulus space itself from the structure of the induced patterns of neuronal activity. The identity of a particular stimulus, then, emerges from a combination of modality (the location and type of activated neurons) and the relationship of its corresponding cell group to all others in an internally represented space. Any necessary assumptions about receptive fields may be regarded as a kind of “universal grammar” [54] that renders stimulus space reconstruction possible.

Recently it has been suggested that sequential replay, as observed in hippocampus and neocortex [55]–[58], may be a mechanism for consolidating sequences of cells that under spatial navigation conditions fire within larger time windows. The sequences reflect groups of cells that co-fire within the same theta cycle during behavior, however, and it is unclear to what extent the precise ordering matters [59],[60]. It is therefore plausible that cell groups may be communicated to cortex during replay events—on a compressed timescale—enabling identification via coincidence detection. This may allow for building representations of space and computing topology in cortex, as these computations require knowledge of the full collection of cell groups.

In summary, we have shown that a surprising amount of information about the structure of stimulus space can be obtained from the combinatorics of cell groups, extracted from noisy population spiking data with a coarse time window. Although we were able to demonstrate the presence of this information constructively, whether and how the brain uses this information remains to be seen. Our results suggest, nevertheless, that combinatorial relationships between groups of cells that fire together could reflect stimulus space structure inside the brain, and may perhaps lead to a general principle of how the brain constructs representations of the outside world.

## Methods

Here we describe how to compute homology groups and construct an internal representation of space from neural spiking data. The starting point for each method is the identification of cell groups. We begin, however, by outlining some basic assumptions about place fields needed for these procedures to work, and a description of the simulated data we used to test our approach.

### Assumptions about Place Fields

(1) Place fields are omni-directional, as is typical in an open field environment, but not on a linear track [21],[61]. (2) Place fields have been previously formed and are stable. (3) The collection of place fields corresponding to observed cells covers the entire traversed environment. (4) The holes/obstacles are larger than the diameters of place fields. (5) Each (connected) component field of a single or multipeaked place field is *convex*. (6) Background activity is low compared to the firing inside the place fields. (7) Place fields are roughly circular and have similar sizes, as is typical in dorsal hippocampus [41],[42].

Although individual electrophysiological recordings can only simultaneously monitor a limited number of cells, it is almost certain that the hippocampus possesses enough place cells for any given environment such that the corresponding place fields cover the entire explored space many times over [44]. The convexity of component fields means that a straight line segment connecting any two points in the field will itself be entirely contained within the field. This is consistent with the observation that individual fields tend to have circular or elliptical shape [21]. Although these are reasonable assumptions about dorsal hippocampal place cells, they pose significant constraints on the quality and quantity of cells in the recording. One may assume, however, that downstream structures in the brain receiving hippocampal output *do* have access to this kind of data. We test our approaches for constructing internal representations and computing homology groups on simulated data that satisfy these criteria.

### Simulated Data

Each environment is an *L*×*L* box, with or without holes in the interior. All length units are with respect to the side length *L* of the box (typically *L*∼1 m). In order for the topology and metric algorithms to work, we of course need the animal to fully explore the environment. In particular, the trajectory must be dense enough to sample the majority of cell groups. For the geometric reconstructions, this is merely a matter of resolution, as sampling fewer cell groups will lead to less precise geometric information. For the topology computations, we need to ensure that the set of all cell groups reveals the full low-order intersection information in order for the low-order homology groups to be accurate. For accurate computation of the *n*th homology group H*_n_*, we need up to (*n*+1)-fold intersections to be detectable via cell groups. If we were only interested in the first homology group H_1_ (this is enough to detect holes/obstacles and distinguish between environments) we need only guarantee that pairwise intersections are accurately reflected—i.e., the trajectory must pass through each pairwise intersection of place fields at least once. However, because we compute homology groups up to H_5_, in order to check consistency of the data with the interpretation as a two-dimensional environment, we have used denser trajectories in our simulations. This would not be necessary if we were only interested in H_0_ and H_1_. Note that a high-order cell group of n cells, signifying an n-fold intersection, implies all lower-order intersections.

#### For topological features

For each of five environments (Figure S1) we generated a smoothed random-walk trajectory, with speed = 0.1 *L*/s (this is 10 cm/s for a 1 m×1 m box), which was constrained to “bounce” off boundaries and stay within the environment. The total duration of each simulated trajectory was 50 minutes. For each of 300 trials, *N* = 70 place fields were generated as disks of radii 0.1 *L* to 0.15 *L*, with radii and centers chosen uniformly at random. In order to ensure place fields covered the environment, centers were chosen initially uniformly at random from uncovered space. Once all space was covered, remaining place field centers were chosen at random from the entire box (Figure S1).

For each place cell in each trial, an average firing rate was chosen uniformly at random from the interval 2–3 Hz. A spike train was generated from the trajectory and corresponding place field as an inhomogeneous Poisson process with constant rate when the trajectory passed inside the place field, and zero outside, so that the overall firing rate was preserved. Because we threshold the number of spikes in each time bin to obtain cell groups, this is equivalent to having somewhat larger non-constant place fields where the firing rate drops quickly below threshold outside the specified radius. Noisy spike trains were created according to the noise percentage **r** (0–10%) as follows. **r**% spikes were deleted from the spike train, and then added back to the spike train at random times, irrespective of position along trajectory, so as to preserve overall firing rate. ‘Shuffled’ data sets were constructed by randomly choosing cells from each of the five environments, and pooling them together to yield population spiking activity that did not come from a single environment.

#### For reconstruction of space

Here we consider a square box environment with no holes. Place fields were generated with radii selected uniformly at random from the interval [0.1 *L*, 0.125 *L*]. This is consistent with the 20–25 cm average place field diameters typically observed for dorsal hippocampal place cells in an environment of scale *L*∼1 m [41],[42]. In the simulations for Figure 4C, place fields sizes were generated from gamma function distributions (shown in Figure 4B) all having peaks at 0.1125 *L* and having minimal place field radius of 0.05 *L*. The location of the peak, the minimum place field size and the standard deviation uniquely determine each gamma distribution. Average firing rates were chosen uniformly at random from the interval 1–3 Hz. The trajectory, locations of place fields, and the spike trains for each place cell were generated as described above. For each total number of cells (*N*_cells_ = 40–140, increasing by 5), we had 60 trials, each with different randomly chosen place fields and inhomogeneous Poisson spike trains.

#### Simulations with multipeaked place fields

In simulations with multipeaked place fields, secondary fields were randomly-generated for the population of multipeaked place cells with the condition that the center of the second field was a distance greater than 0.5 *L* away from the center of the first randomly-generated field. This was to guarantee that pairs of fields for double-peaked cells were sufficiently well-separated to allow detection of separate fields via cell groups (see Figure S5 and Figure S7). A higher density of coverage by place fields would allow the distance between multiple fields of the same cell to be smaller, approaching the minimal separation required for the fields to be disconnected. The radii for the component fields in double-peaked place fields were drawn independently from the same distributions of radii used for single-peaked place fields. All other aspects of the simulations with multipeaked place fields were the same as for simulations with only single-peaked place fields.

### Identification of Cell Groups

We define a *cell group* as a group of place cells that collectively fire within a two theta-cycle (250 ms) time window. To determine the full set of cell groups that become activated as the animal traverses the environment, we first bin population spike trains into 2-theta-cycle time bins. A certain subset of cells fires in each time bin, and we use these subsets to determine the cell groups. Because there is some probability that a given place cell will fire outside its place field, we impose a threshold on firing rates in order to determine the group of cells that fired significantly above baseline for each bin. Each resulting cell group can then be assumed to correspond to a particular intersection of place fields.

We first divided population activity into a set of population vectors, i.e., vectors in **R** *^N^* ^cells^ with firing rates for each cell in a given time bin. In order not to miss any cell groups due to the arbitrary choice of where bins start and end, the binning time windows were then shifted to have a total of five different starting positions (eight for topology), equally spaced within two theta-cycles, so that each spike contributed to five population vectors. All population vectors were pooled and thresholded as follows. For each cell, the firing rate in a particular population vector was considered significant if it was at least 6 times the average firing rate for that cell. Each population vector thus yielded a cell group, consisting of all cells firing significantly above baseline in a particular time bin. The thresholding is what renders the topology and reconstruction of space procedures fairly robust to noise in the spike trains.

### Extraction of Topological Features

Here we describe how to compute the homology groups of a given environment from the collection of all cell groups that are active in the environment.

#### Some mathematical preliminaries

We use a few standard mathematical objects that are uncommon in the neuroscience literature. Here we give brief descriptions of these objects; see Text S1 for rigorous definitions. A *simplicial complex* is a set of vertices and simplices (simplices are *n*-dimensional triangles: points, line segments, triangles, tetrahedra, etc.). An *abstract simplicial complex* is a set with a set of subsets satisfying similar properties as simplices. We will use it as a combinatorial object that keeps track of intersection information revealed by cell groups. Roughly speaking, *homology groups* [38],[45],[62] count the number of “holes” of various dimensions in a given topological space. The dimensions β*_i_* of the homology groups H*_i_* are called *Betti numbers*. The 0^th^ Betti number β_0_ counts the number of connected components in a space, while β_1_ counts the number of holes that can be bordered by a closed 1-dimensional contour. Higher Betti numbers β*_i_*, *i*>1, count the number of “holes” in higher dimensions. There are many definitions of homology groups that can be shown to be equivalent in most cases of interest [38]. We use *simplicial homology groups*. Simplicial homology groups are defined for any topological space which can be subdivided into a simplicial complex; they are also defined for abstract simplicial complexes. In other words, the definition applies to the two-dimensional spatial environments explored by the animal, as well as to the high-dimensional abstract simplicial complexes we obtain from population spiking data.

#### Computation of homology groups from cell groups

The set of all cell groups for a complete data set naturally yields an abstract simplicial complex. Each cell is a vertex, and each group of *n* cells yields an (*n* – 1)-dimensional face (Figure 1B). A 1-dimensional face is an edge, a 2-dimensional face is a triangle, a 3-dimensional face is a tetrahedron, and so on. Assuming place fields are *convex*, a deep theorem in algebraic topology [38] implies that the homology groups of this simplicial complex are equal to the homology groups of the underlying space (see Text S1). We can thus distinguish between different environments by computing their homology groups from population spiking data alone. For our two-dimensional flat environments, β_0_ is always 1 and higher Betti numbers (β*_i_*, *i*>1) all vanish. (If the animal were exploring the entire surface of a ball, however, we would expect β_2_ = 1.) The 1^st^ Betti number β_1_, on the other hand, is different for each of the five environments, matching the number of holes in each.

To compute homology groups for the very large and high-dimensional simplicial complexes defined by cell groups, we use an algorithm from computational algebraic topology implemented for the GAP software package [63],[64]. The algorithm relies exclusively on standard linear algebra, and is thus in principle realizable by a simple neural network. We computed the first five homology groups H_0_,…,H_4_, and declared a trial to be ‘correct’ when all Betti numbers matched what was expected for the environment: β_0_ = 1, β_1_ = number of holes, and β*_i_* = 0 for *i*>1. A trial was deemed to be ‘incorrect’ if at least one of the five computed Betti numbers did not match.

#### “Refinement” step for multipeaked place cells

In the previous analysis, the convexity of place fields was needed such that the open cover (see Text S1) associated to the set of all place fields satisfied the properties necessary for the theorem to hold. If the data includes place cells having multipeaked place fields, we need to assign an open set for each connected component of the place field. Fortunately, multiple fields can easily be detected from the set of all cell groups, by identifying connected components in the induced subgraph of neighborhood relationships between cell groups (see Figure S7). By assigning a distinct open set for each component of this graph, we can then build the simplicial complex and compute homology groups exactly as if each field corresponded to a different cell. The added step to the topology algorithm can be thought of as making the minimal possible *refinement* [45] of the open cover, defined by the (unknown) place fields, such that the theorem again holds. A *refinement* of an open cover is a new cover such that each set in the new cover is fully contained in an open set of the old one. In order to detect the connected components of a graph defined via an adjacency matrix, we used the standard Dulmage-Mendelsohn matrix decomposition, implemented in the Matlab routine ‘dmperm’.

### Internal Reconstruction of Space

Here we describe the construction of an internal representation of the environment from the collection of all cell groups that are active in that environment. This can be summarized in two steps: (i) construction of a graph, containing a vertex for every cell group and an edge between neighboring cell groups, and (ii) construction of a distance matrix (or metric) containing distances between any two cell groups. In order to verify that the internal representation is faithful to the geometry of the external space, we computed the average error on pairwise distances between points in the external space as estimated using the metric for the internal representation. To further validate that the full geometry is accurately reflected in the internal representation, we used multidimensional scaling (MDS) to embed the graph in two-dimensional Euclidean space in a way that best preserves the metric on cell groups. This enables comparison of the full geometries by visual inspection and by computation of the *mismatch* (see below).

#### Regions represented by cell groups

Each cell group defines a point, or small region in space contained in the intersection of the corresponding place fields, but not in any higher order intersection (as this would correspond to adding additional cells to the cell group). Mathematically, if *C_k_*⊆{1,…,*N*_cells_} denotes a cell group with *k* cells, and 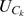 is the intersection (as a subset of the environment) of the corresponding place fields, then the region of space corresponding to the cell group *C_k_* is given by 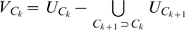. For example, the colored regions in Figure 1C and Figure 3B all correspond to subsets of the form 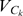. Similarly, the black dots in Figure 3C and 3D, each corresponding to a cell group *C_k_*, represent regions of space of the form 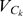, not pure intersections 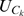.

#### Dissimilarity index

The distances between any two cell groups are computed via a dissimilarity index *μ_k_* on neighbors (cell groups that differ by just one cell). For each total number *N*_cells_ of place cells, we estimated *μ_k_* empirically by computing the average distances between the centers of adjacent intersection regions 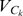 and 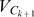 for 30 randomly-generated sets of place fields having uniform radii (*r* = 0.1) covering the environment. We normalized the index by fixing the largest value *μ*_1_ = 1. Note that for each value of *N*_cells_, *μ_k_* depends only on the order *k* of the smaller cell group. Empirically computed values of *μ_k_* for differing numbers of cells are shown in Figure S2A. In principle, *μ_k_* should be derivable from basic geometry; we find that it is well approximated by the formula 
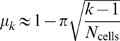
, for 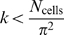 (Figure S2B). Although this formula was obtained assuming all place fields have exactly the same size, we have used the same formula for place fields of varying radii, regardless of the particular distribution (uniform or gamma) being considered.

#### Distance matrix (metric on cell groups)

Given a collection of cell groups, we obtain a distance matrix (or metric) containing distances between any two cell groups as follows. We first construct a graph whose vertices are cell groups, and whose edges are given by neighboring pairs of cell groups (Figure 3C). To each edge between neighbors of degrees *k* and *k*+1 we assign the weight *μ_k_* (Figure 3D). A *path* is a sequence of edges connecting two vertices in the graph; the length of a particular path is given by summing the weights along its edges. The distance between any two cell groups (vertices in the graph) can then be defined as the length of a shortest path between those points (Figure 3D). We use Johnson's ‘all shortest paths’ algorithm [65], implemented for Matlab in [66], to construct a distance matrix with distances between each pair of cell groups. Note that this yields a *metric* in the strict mathematical sense, as it is positive definite, reflexive, and satisfies the triangle inequality. Finally, we add a 0–1% random noise jitter to the entries of the distance matrix, to ensure that the MDS method we later use (below) does not encounter any degeneracies due to multiple entries of the matrix being exactly equal.

#### Mapping between spaces

Points in the original space are mapped into the internal representation as follows. Assuming place fields cover the environment, any point in the original space lies in a particular intersection region 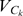, and is expected to activate the cell group *C_k_*, where *k* is the number of cells (equivalently, the order of intersection). We can thus identify points in the original space with the corresponding cell groups (vertices) in the internal representation. In cases where the number of cells was not sufficient to completely cover the environment, uncovered points are mapped to the internal space using the nearest cell group. For a given point *p* in the environment, we denote the corresponding cell group as *C*(*p*), regardless of the number of cells it contains.

#### Pairwise error

In order to quantitatively assess the quality of the internal representation, we compared distances between pairs of points (*p*,*q*) in the original environment to the distances between their corresponding cell groups *C*(*p*) and *C*(*q*) in the internal representation. For a given trial, we computed the *pairwise error* as the average value of ∥*p*−*q*|−*d*(*C*(*p*),*C*(*q*))|, where *d* is the (renormalized) constructed metric on cell groups, and the average is taken over all pairs of points (*p*,*q*) coming from the grids shown in Figure S3. Because the scale for the metric on cell groups was set by the convention *μ*_1_ = 1, we multiplied the constructed metric for the internal representation by an overall constant such that the mean pairwise distance computed using *d* matched the mean pairwise distance in the external environment; this ensured that differences in overall scale would not contribute to the pairwise error. The average pairwise error for 60 trials, as a function of the total number of place cells, is shown in Figure 4.

#### Embedded internal representation

Given a distance matrix for a collection of points, and a specified dimension, a non-metric MDS algorithm [43] arranges the points in Euclidean space so as to best preserve the ordering of the distances in the distance matrix. (In other words, nearby points will be mapped close together and far away points will be kept far away, though actual distances may be distorted.) We use the Matlab implementation ‘mdscale.’ This enables us to visually assess how accurately the internal representation reflects the full external geometry, beyond just pairwise distances between points. Lines and trajectories in the original space can be mapped into the embedded internal representation by “connecting the dots” between the images of their points (see Figure 5A).

#### Comparison to original space, alignment, and mismatch

The output of MDS is only unique up to a Euclidean transformation (rotation and translation). Moreover, the overall scale in our distance matrix is arbitrary, as we normalized our dissimilarity index on neighbors such that only relative distances mattered. In order to compare the raw MDS output to the original space we must therefore “align” the internal representation properly. We do this by finding the optimal affine transformation (rotation, translation and scaling) that minimizes the distances between points in the original space and their images in the internal representation space.

An affine transformation is a transformation of the form
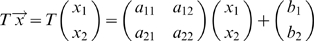
parameterized by six numbers (*a*_11_,*a*_12_,*a*_21_,*a*_22_,*b*_1_,*b*_2_). This amounts to translating (2 parameters), rotating (1 parameter) and scaling in two independent directions (3 parameters, the third is the angle between directions). We find an optimal affine transformation *T* relating the raw MDS output to the external space by minimizing a function of six variables
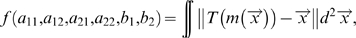
where 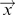 is a point in the external space and 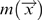 is its image after mapping to the internal space. The double integral is taken over the entire square area of the external environment, and was computed by summing over a grid of 150×150 points. The optimization was performed using ‘fminsearch’ in Matlab. The resulting optimal transform was then used to align the raw MDS output to better match the coordinates on the original space. Figure S4 shows raw MDS outputs (left column) and corresponding aligned versions (middle column).

After alignment, we can evaluate the quality of the representation by computing its “mismatch” with the original space. A fine grid of points (150×150) in the original space is mapped to the aligned internal space, and the distances 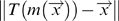 between grid points and their images are computed, as a fraction of the box side length *L*. The average of all of these distances is called the *mismatch*.

Note that the alignment procedure, which *does* require the use of place fields and independent position information, is only necessary for computing the mismatch—i.e., to quantify how well the embedded internal representation directly compares to the external space. This is because the particular coordinate systems we choose to parameterize the internal and external spaces are completely arbitrary, and must be shifted, scaled and rotated to match. The brain does not need to perform either MDS or “alignment”; it need only track position with respect to its own, internally constructed representation of space.

## Supporting Information

Figure S1 Five different environments used in simulations. The trajectories (green) were generated using a smooth random walk. Sample place fields for one trial per environment are depicted as gray circles. The holes/obstructions can be seen as white rectangles not covered by the trajectory.
(7.10 MB EPS)

Figure S2An approximate formula for the index *μ_k_*. (A) The dissimilarity index *μ_k_* on neighboring cell groups, for different numbers of cells, computed empirically (see Methods). (B) Comparison between empirically computed *μ_k_* (black traces) and the formula
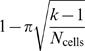
(red traces) for various values of *N*_cells_. The value of *μ_k_* for the very highest occurring *k* in each case is not displayed, as very few such intersections occurred, rendering the empirical estimate unreliable.(1.06 MB EPS)

Figure S3 Two grids used for computing pairwise distances. We considered pairwise distances between all possible pairs of points (*p*,*q*), where *p* is a point on a fine 100×100 grid (gray dots) and *q* is a point on a coarse 4×4 grid (red dots) in the square environment. (*q* is taken from a coarse grid to reduce the total number of pairs from 10^8^ to a more computable 1.6*10^5^.) For each trial, the pairwise error was computed as the average value of ∥*p*−*q*|−*d*(*C*(*p*),*C*(*q*))|, where *C*(*p*) and *C*(*q*) denote cell groups corresponding to points *p* and *q*, respectively, and *d* was the constructed metric on cell groups. This provides a measure for the quality of an internal representation constructed from cell groups.
(3.69 MB EPS)

Figure S4 Internal space reconstructions for increasing numbers of place cells. The original environment (bottom right) with three sample place fields. A coarse grid (red and orange lines) is used for visual comparison with the reconstructed spaces, as in Figures 5 and 6. Black and colored dots correspond to cell groups, as in Figure 3C and 3D, with colors representing cell groups containing the three sample place cells. Raw MDS outputs (left column) for the internal reconstructions of space have arbitrary scaling and orientation; aligned versions (middle column) can be used to compute the mismatch (see Methods). Mismatch improves with increasing numbers of place cells.
(4.97 MB EPS)

Figure S5 Cell groups containing place cells with multipeaked place fields. Black dots correspond to cell groups for the reconstructed space shown in Figure 6B. The reconstruction was obtained from the simulated activity of 127 cells, 13 of which had multipeaked place fields. For each of the 13 place cells with multipeaked place fields, all cell groups containing that place cell are plotted in red (plots 1–13). Cell groups for a pair of single-peaked place fields are also shown (plots 14, 15).
(11.61 MB EPS)

Figure S6 Multipeak pairwise error and mismatch for coverage by 90 and 140 fields. The presence of place cells with multipeaked place fields does not affect the performance of the metric reconstructions so long as the double fields are themselves fully covered by other place fields, in which case the corresponding cell groups are fully disambiguated by other cells. (A,B) For a total coverage by only 90 fields (including double fields for multipeaked cells), both the pairwise error and mismatch have increasing mean and variance for increasing percentages of multipeaked cells. This is because 90 randomly-located fields for the given range of radii (shaded region, dashed line indicates mean place field radius) are not enough to double-cover the environment. (C,D) The environment is fully double-covered with 140 fields. Accordingly, there is no significant decrease in performance for increasing percentages (up to 11%) of multipeaked place fields. ((D) is the same as Figure 6C.)
(0.92 MB EPS)

Figure S7 Place cells with multipeaked place fields can be detected from cell groups. Overlapping circles (middle) illustrate an example place field configuration for an environment with no holes. Cell 8 has a double-peaked place field, consisting of two disconnected regions (shaded gray areas). A graph, as in Figure 3, can be constructed from the correspondingly activated cells groups (not shown). For each cell, there is an associated subgraph induced by restricting only to cell groups (vertices) that contain the given cell. The subgraph associated to cell 8 (left) has two connected components, indicating that this place cell has a place field with two disconnected firing fields. In contrast, the subgraph for cell 3 (right) is connected, as are the subgraphs for all other place cells (1–7) in this example (not shown). Note that identification of multipeaked place fields requires that they be entirely covered by other fields, as is the case of cell 8. For this reason, we must have enough cells to double-cover the environment by place fields in order to guarantee that we can identify place cells with multipeaked fields and disambiguate disconnected fields.
(0.61 MB EPS)

Text S1 Supplementary Text
(0.11 MB PDF)
