# Supplementary material for: Cell Groups Reveal Structure of Stimulus Space
Source: PLoS Comput Biol. 2008 Oct 31;4(10):e1000205. doi: 10.1371/journal.pcbi.1000205 (PMC2565599; doi:10.1371/journal.pcbi.1000205)
Supplement: Text S1 — Supplementary Text (0.11 MB PDF) [file pcbi.1000205.s008.pdf]

Supplementary Text

to accompany "Cell Groups Reveal Structure of Stimulus Space"

Carina Curto & Vladimir Itskov

2008

Here we present a brief exposition of some material from algebraic topology that we use in our methods. We include it for completeness, as it may not be familiar for many readers. In particular, we define simplicial complexes, simplicial homology groups, and state the theorem cited in the Results section. See [Bott and Tu, 1982, Ewald, 1996, Hatcher, 2002] for more details.

1 Preliminary definitions

Convexity. A set $C \subset \mathbb{R}^n$ is called *convex* if, for all $x, y \in C$, the straight line segment between x and y is contained in C:

$$\{tx + (1-t)y \mid 0 \leq t \leq 1\} \subseteq C.$$

Convex hull. The *convex hull* of a set of points $x_1, ..., x_r \in \mathbb{R}^n$ is the set of points

$$\mathrm{conv}(\{x_1, ..., x_r\}) := \{\sum_{i=1}^{r} t_i x_i \mid \sum t_i = 1 \text{ and } t_i \geq 0 \text{ for all } i\}.$$

For example, the convex hull of two points is the straight line segment between them, the convex hull of three non-colinear points in a plane is a triangle, and the convex hull of four non-coplanar points in $\mathbb{R}^3$ is a tetrahedron.

Simplex. A *simplex* of dimension k is the convex hull of a set of points $S = \{x_1, ..., x_{k+1}\}$, provided the convex hull is k-dimensional. The vertices of

the simplex are the points in S. The convex hull of a subset of $m+1$ points in S is called an m-dimensional *face* of the simplex. Vertices are 0-dimensional faces, edges are 1-dimensional faces, triangles are 2-dimensional faces, and so on. The empty set is also considered to be a face of every simplex, by convention.

Simplicial complex. A *simplicial complex* K is a set of simplices such that (i) any face of a simplex in K is also in K, and (ii) the intersection of any two simplices $\sigma_1, \sigma_2 \in K$ is a face of both σ_1 and σ_2. The dimension of a simplicial complex is the dimension of its highest-dimensional simplices.

Abstract simplicial complex. An *abstract simplicial complex* is a set $\{1, ..., N\}$ with a set K of subsets such that if $Y \in K$ and $X \subset Y$ then $X \in K$. In other words, every subset of a subset in K is also included in K.

Every simplicial complex is an abstract simplicial complex, where the elements of the set $\{1, ..., N\}$ correspond to labelled vertices, and the set of subsets K corresponds to simplices (together with their faces, which are also simplices). Conditions (i) and (ii) for a simplicial complex guarantee that every subset of a subset in K is also in K.

2 Simplicial homology with real coefficients

There are many definitions of homology groups, and these can be shown to be equivalent for many spaces of interest [Hatcher, 2002]. We need only compute homology for two kinds of topological spaces: simplicial complexes (obtained from cell groups) and our two-dimensional environments. Simplicial homology with real coefficients is comparatively easy to define, and suffices for our purposes.

Chains. Let $K = \{\sigma_i^k\}$ be a simplicial complex with simplices σ_i^k, where k denotes the simplex dimension. A simplicial k-*chain* is a formal sum of k-dimensional simplices

$$\sum_{i=1}^{N_k} c_i \sigma_i^k,$$

where $c_i \in \mathbb{R}$ and N_k is the number of k-dimensional simplices in K. Let $\mathcal{C}_k$ denote the group of all possible k-chains on K. Since our coefficients are in $\mathbb{R}$, $\mathcal{C}_k$ is just the vector space $\mathbb{R}^{N_k}$.

Boundary operator. Any k-simplex can be represented as an ordered set of vertices

$$\sigma^k = \langle v_0, v_1, ..., v_k \rangle,$$

where exchanging any two vertices $v_i \leftrightarrow v_j$ in the ordering produces a change in sign: $\sigma^k \to -\sigma^k$.

The *boundary operator* ∂, acting on simplices, is defined as

$$\partial(\sigma^k) = \sum_{i=0}^{k} (-1)^i \langle v_0, v_1, ..., \hat{v}_i, ..., v_k \rangle,$$

where the simplex $\langle v_0, v_1, ..., \hat{v}_i, ..., v_k \rangle$ is the $(k-1)$-dimensional face of σ^k obtained by deleting its i-th vertex. This definition can now be extended linearly to chains, so that $\partial : \mathcal{C}_k \to \mathcal{C}_{k-1}$, with

$$\partial \left(\sum_{i=1}^{N_k} c_i \sigma_i^k \right) = \sum_{i=1}^{N_k} c_i \, \partial(\sigma_i^k).$$

Note that since $c_i \in \mathbb{R}$, the boundary operator is a linear transformation $\partial : \mathbb{R}^{N_k} \to \mathbb{R}^{N_{k-1}}$, where the simplices σ_i^k represent basis elements for $\mathbb{R}^{N_k}$.

Cycles and boundaries. Consider the composition of two boundary operators

$$\mathcal{C}_{k+1} \xrightarrow{\partial_{k+1}} \mathcal{C}_k \xrightarrow{\partial_k} \mathcal{C}_{k-1}.$$

For a given $(k+1)$-simplex, this yields

$$\begin{aligned}
\partial_k \, \partial_{k+1}(\sigma^{k+1}) &= \sum_{i=0}^{k+1} (-1)^i \, \partial \langle v_0, v_1, ..., \hat{v}_i, ..., v_{k+1} \rangle \\
&= \sum_{i=0}^{k+1} (-1)^i \left(\sum_{j<i} (-1)^j \langle v_0, v_1, ..., \hat{v}_j, ..., \hat{v}_i, ..., v_{k+1} \rangle + \right. \\
&\quad \left. \sum_{j>i} (-1)^{j-1} \langle v_0, v_1, ..., \hat{v}_i, ..., \hat{v}_j, ..., v_{k+1} \rangle \right) \\
&= 0.
\end{aligned}$$

Thus the composition $\partial_k \circ \partial_{k+1} \equiv 0$ for all k. (It is often denoted $\partial \, \partial$ when the spaces $\mathcal{C}_k$ are unambiguous.) For example, on the triangle $\langle v_0 v_1 v_2 \rangle$, we

have

$$\begin{aligned}\partial\,\partial\langle v_0, v_1, v_2\rangle &= \partial(\langle v_1 v_2\rangle - \langle v_0 v_2\rangle + \langle v_0 v_1\rangle) \\ &= \langle v_2\rangle - \langle v_1\rangle - (\langle v_2\rangle - \langle v_0\rangle) + \langle v_1\rangle - \langle v_0\rangle = 0.\end{aligned}$$

The identity $\partial\,\partial \equiv 0$ implies that

$$\operatorname{im}\partial_{k+1} \subset \ker\partial_k\,.$$

Elements in $\ker\partial_k$ are called *cycles*, and elements in $\operatorname{im}\partial_{k+1}$ are called *boundaries*. An element of $\ker\partial_k$ which is a cycle but is *not* a boundary corresponds to a "hole" of dimension k in the simplicial complex. For example, if a simplicial complex contained the 1-cycle

$$\langle v_1 v_2\rangle - \langle v_0 v_2\rangle + \langle v_0 v_1\rangle \in \ker\partial_1 \subset \mathcal{C}_1$$

(which corresponds to adding directed edges along the triangle with vertices v_0, v_1 and v_2), but did *not* contain the triangle $\langle v_0 v_1 v_2\rangle$, then this 1-cycle would represent a 1-hole in the simplicial complex.

Homology groups. Simplicial homology groups are designed to detect holes of various dimensions in a simplicial complex. Many topological spaces can be represented as simplicial complexes, so the definition can be applied widely.

The kth *homology group* H_k of the simplicial complex K is defined as the quotient

$$H_k(K) = \frac{\ker\partial_k}{\operatorname{im}\partial_{k+1}}.$$

In other words, it consists of cycles in $\mathcal{C}_k$ modulo boundaries. Again, because we are dealing with real coefficients, $\mathcal{C}_k \cong \mathbb{R}^{N_k}$, the boundary operators ∂_k are linear transformations, and the homology groups H_k are just vector space quotients.

Betti numbers. Often one is only interested in extracting the number of holes in each dimension of a topological space. The number of k-holes is given by the dimension of the kth homology group, and is denoted

$$\beta_k = \dim(H_k(K)).$$

These integers are called the *Betti numbers* of the space K.

Remarks. Homology groups (and Betti numbers) are examples of *topological invariants*. In other words, they are preserved under *homeomorphisms* (continuous maps with continuous inverses between two topological spaces). Homology groups are also invariant under *homotopy equivalence*, which relates spaces that may be of different dimensions, but have the same numbers of "holes" in each dimension. For example, a two-dimensional annulus is homotopy equivalent to a one-dimensional circle, although these spaces are topologically distinct. (See [Hatcher, 2002] for more details.)

Homology groups for other topological spaces. We have defined simplicial homology for simplicial complexes. The definition extends, however, to any topological space X which can be represented as a simplicial complex. This is done by finding a *triangulation* of the space – i.e. a simplicial complex K which is homeomorphic to X, together with a homeomorphism $h : K \to X$. Because K and X are homeomorphic, the homology of X equals the homology of K.

For example, in the case of our two-dimensional environments, a triangulation can be obtained by sub-dividing the space into triangles. This yields a two-dimensional simplicial complex (quite different from the one we obtain from cell groups on the same space!) that can be used to directly compute the homology of the space.

3 Statement of the theorem

The main algebraic topology result we use states that the homology of a topological space is equal to the homology of a simplicial complex constructed from an open cover of that space [Hatcher, 2002]. A variant, applicable only for the first homology group, can be found in [Bott and Tu, 1982, Theorem 13.4, p. 148]. We will state the theorem with the minimum generality necessary for our methods, so as to make its application as transparent as possible. First we need a couple of definitions.

Open cover. An open cover of a topological space X is a set of open sets $\mathcal{U} = \{U_\alpha\}$ such that, for each $x \in X$, $x \in U_\alpha$ for some α.

Nerve of an open cover. To each open cover $\mathcal{U} = \{U_\alpha\}$ of a given space X we can associate a simplicial complex $N(\mathcal{U})$ called the *nerve* of $\mathcal{U}$. This has

a vertex v_α for each U_α, and a k-dimensional simplex $\langle v_{\alpha_1}, ..., v_{\alpha_k} \rangle$ for every set of corresponding U_α's that have nonempty intersection.

Theorem. Let $\mathcal{U} = \{U_\alpha\}$ be an open cover of a compact space $X \subset \mathbb{R}^n$, such that every set U_α is convex. Then the homology of X is equal to the homology of the nerve $N(\mathcal{U})$. In particular, X and $N(\mathcal{U})$ have exactly the same Betti numbers.

In our case, the space X is the two-dimensional environment explored by the animal, and the set of place fields forms an open cover $\mathcal{U}$ of X. The nerve of the cover $N(\mathcal{U})$ is precisely the simplicial complex we construct from cell groups. Because each place field is assumed to be convex, the theorem implies that the homology of this simplicial complex is equal to the homology of the underlying space. Since we obtain the simplicial complex purely from cell groups, this means we can compute the homology of the environment using only population spiking activity. Note that, in practice, our simulated data do not always yield a perfectly accurate collection of cell groups; this is due to the stochastic nature of the Poisson process used to simulate spiking activity, and also to the added noise. This is why some of our trials are 'incorrect,' even though the theorem guarantees the success of the computation.

The theorem is an easy consequence of Corollary 4G.3 in [Hatcher, 2002], which is itself a greatly simplified version of a much more general result.

Corollary 4G.3 [Hatcher, 2002, p. 460]. If $\mathcal{U}$ is an open cover of a paracompact space X such that every nonempty intersection of finitely many sets in $\mathcal{U}$ is contractible, then X is homotopy equivalent to the nerve $N(\mathcal{U})$.

The theorem follows because every compact space is paracompact, the convexity of the sets in $\mathcal{U}$ guarantees that all nonempty finite intersections are contractible, and because homotopy equivalence between two spaces implies that they have the same homology groups.

4 Computing homology of simplicial complexes

Our simplicial complexes obtained from cell groups are very large and high-dimensional; it is impossible to compute the homology by hand. Instead, we use an algorithm from computational algebraic topology, implemented for

the GAP software package. It is available at
`http://www.cis.udel.edu/~linbox/gap.html`.

The computations can also be done with Plex, a recently released package for Matlab, available at `http://comptop.stanford.edu/programs/`.

References

[Bott and Tu, 1982] Bott, R. and Tu, L. W. (1982). *Differential forms in algebraic topology*, volume 82 of *Graduate Texts in Mathematics*. Springer-Verlag, New York.

[Ewald, 1996] Ewald, G. (1996). *Combinatorial convexity and algebraic geometry*, volume 168 of *Graduate Texts in Mathematics*. Springer-Verlag, New York.

[Hatcher, 2002] Hatcher, A. (2002). *Algebraic topology*. Cambridge University Press, Cambridge.
